# Zinc recovery from metallurgical slag and dust by coordination leaching in NH_3_–CH_3_COONH_4_–H_2_O system

**DOI:** 10.1098/rsos.180660

**Published:** 2018-07-11

**Authors:** Aiyuan Ma, Xuemei Zheng, Song Li, Yihong Wang, Shan Zhu

**Affiliations:** School of Chemistry and Materials Engineering, Liupanshui Normal University, Liupanshui 650093, People's Republic of China

**Keywords:** ammonium acetate, metallurgical slag and dust, coordination leaching, zinc extraction

## Abstract

Metallurgical slag and dust (MSD) from lead and zinc smelting, steel dust and galvanized steel scrap are important secondary sources of zinc and other valuable metals. This paper describes the production feasibility and rationality of a cleaner zinc recovery process using MSD and a hydrometallurgical method. It was found that the addition of CH_3_COONH_4_ to a NH_3_–H_2_O system promotes zinc extraction, and 83.76% of zinc could be dissolved and recovered from the MSD under the following conditions: total ammonia concentration of 5 mol l^−1^, stirring speed of 300 r.p.m., ammonia/ammonium ratio of 1 : 1, solid/liquid ratio of 1 : 5, leaching temperature of 25°C and a leaching time of 60 min. A leaching kinetic study indicates that the leaching process is controlled by the diffusion and interface transfer and that the reaction apparent activation energy is 22.66 kJ mol^−1^. Fourier transform infrared spectroscopy and electrospray ionization mass spectrometry analysis showed that zinc can combine with the carboxylate anion to form Zn complexes such as [Zn_2_(Ac)_3_(NH_3_)_2_]^+^. Zn_2_SiO_4_, ZnS and ZnFe_2_O_4_ in NH_3_–CH_3_COONH_4_–H_2_O system did not disappear according to X-ray diffraction analysis for leaching residue.

## 1. Introduction

Currently, the main source of zinc metal is the traditional zinc resource—zinc sulfide ores. However, as the zinc consumption increases and the zinc sulfide ore grades deteriorate, the gap between supply and demand has become a major problem for the zinc industry [1,2]. At the same time, large quantities of metallurgical slag and dust (MSD) are constantly produced during the production of steel, lead and zinc smelting, which causes serious pollution owing to the waste residue. A zinc content that is over the prescribed limit might have a negative impact on blast furnaces, i.e. it might cause damage to refractory materials, shorten campaign life, cause operation difficulties and decrease the blast furnace productivity [3–5]. Lead and zinc smelting slag contains several poisonous elements such as arsenic (As), cadmium (Cd) and chromium (Cr), which makes it hazardous and unacceptable for stockpiling or landfilling [6–8]. The zinc extracted from MSD has potential economic benefits in recycling, reuse, exploitation and application of solid waste pollutants [9–12]. Therefore, the development of new technologies to produce zinc from MSD has become an important research topic in recent years.

The various solid wastes recovered from steel plants are mainly obtained from sludge, dust and slag. MSD is known to be hazardous and adversely affects the environment; it also has complex structures and contains various metal elements, carbonaceous compounds, chlorides and gangue minerals (silicon, calcium and magnesium) [13–16].

Currently, the hydrometallurgical method is the most common method of zinc extraction, and it involves leaching, purification and electrolysis [17]. In the leaching process, sulfuric acid is the most common leaching agent. However, for MSD, especially MSD that has a high content of iron, chlorides and gangue minerals, excessive acid consumption and a complex purification process are of great concern [18,19].

Nevertheless, the selective extraction of zinc can be achieved through alkaline leaching, as the metal zinc passes into solution, while the iron, gangue minerals, chlorides and fluorides remain in the solid residue [20,21]. The interaction between RCOO^−^ and zinc from different types of minerals has been investigated in previous research works [22,23]. The addition of NTA (nitrilotriacetic acid, N(CH_2_COOH)_3_) to the NH_4_Cl–NH_3_ solutions promotes the conversion of the [Zn(NH_3_)_4_]^2+^ complexes and [Zn(NTA)_2_]^4−^ complexes into the more stable [Zn(NTA)(NH_3_)_2_]^−^ complexes, which further improves the leaching rate of low-grade zinc oxide ores [24]. Ejtemaei [2] reported that species such as RCOO^−^ exist in the basic region (pH = 10.0), and the maximum adsorption of oleic acid (CH_3_(CH_2_)_7_CH=CH(CH_2_)_7_COOH) on smithsonite may be attributed to the interaction between RCOO^−^ and zinc on the smithsonite surface. It can be assumed that the adsorption of oleic acid takes place via an ion exchange mechanism. Steer [25] discusses the mechanisms of extraction for organic carboxylic acids such as malonic, acrylic, citric, acetic, oxalic and benzoic acids in greater detail. The results suggest that the zinc extraction from blast furnace dust slurry can be well explained by substituent group effects and the Lewis acid/base theory.

Furthermore, ammonium ions (NH_4_^+^) and carboxylate anions (RCOO^−^) play an important role in the dissolution process of ZnO and help in the extraction of zinc from the MSD in an economical way.

To solve the problem and realize the use of secondary zinc resources through recycling, a clean zinc production process using NH_3_–CH_3_COONH_4_ as a leaching agent has been developed.

## 2. Material and methods

### 2.1. Experimental materials

**Figure 1.**
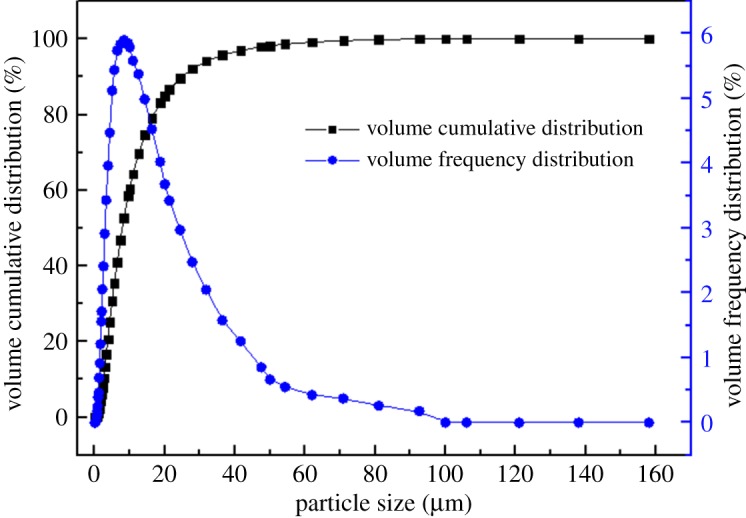
Particle size distribution curves of MSD sample.

The MSD used was supplied by a resource recycling company located in Yunan, China. The sample used consisted of large quantities of MSD including the slag from a lead and zinc smelting plant and industrial solid wastes (sludge, dust and slag) from an ironmaking and steelmaking process. A laser particle size analyzer (JL-1177, Chengdu Jingxin Powder Analyse Instrument Co., Ltd, China) was used to rapidly determine the characteristic parameters of the particles and particle size distribution of the MSD sample. The particle size analysis of a representative MSD sample is presented in figure 1. The dust particles were fine with an average size (d50) of 7.885 µm and 98% of the particles were smaller than 47.912 µm. It can be observed that the majority of the particles were within the 2–50 μm size range.

**Figure 2.**
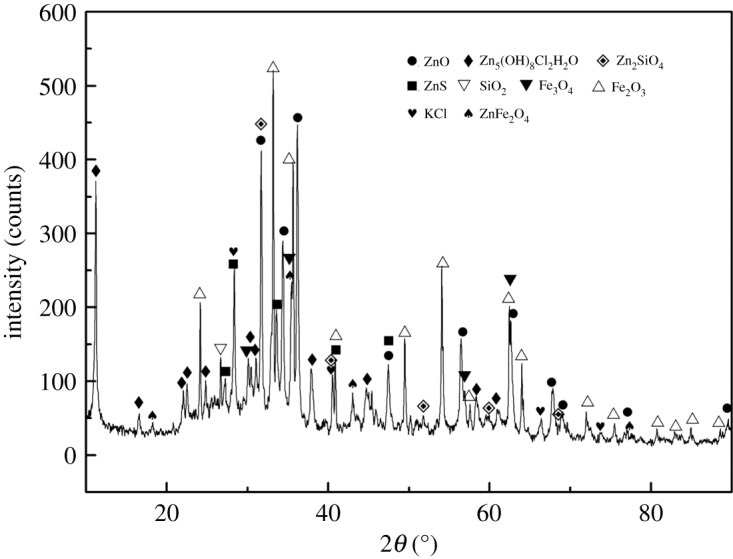
XRD pattern of the MSD.

The mineralogical (phase) composition of the MSD samples was determined by X-ray diffraction (XRD) analysis (Rigau, TTR-III) in the 2*θ* scale using Cu K*α* radiation (*λ* = 1.54056 Å, at 40 kV and 200 mA) at the scanning rate of 4° min^−1^ and varying from 10° to 90°. The main chemical composition and XRD pattern of the samples are presented in table 1 and figure 2, respectively. Zn, Fe, C, Si, Pb, Ca, Mg, Al, S, In and Bi were found to be the major elements in the MSD sample in the present study, and the zinc content in the MSD sample was as high as 24.74%. In addition, it was difficult to concentrate on the zinc using the traditional zinc smelting method owing to the high content of alkaline gangues (5.24%, CaO + Mg) in the MSD sample. Figure 2 shows the MSD sample used in this work, which was mainly composed of ZnO, Zn_5_(OH)_8_Cl_2_H_2_O, Zn_2_SiO_4_, ZnS, Fe_2_O_3_, Fe_3_O_4_ and ZnFe_2_O_4_, while SiO_2_ was identified as the main gangue component.

**Table 1.** Main chemical composition of MSD (mass fraction, %).

element	Zn	Fe	C	Si	Pb	S	Al_2_O_3_	CaO	Mg	In (g/t)	Bi
weight (mass %)	24.74	21.66	9.14	2.66	1.13	1.39	2.22	4.10	1.14	354	0.97

At the same time, to investigate the effect of particle size distribution on the zinc leaching rate, the MSD samples were sieved and divided into seven particle sizes using standard Tyler sizes. The proportion of zinc content and weight for different particle sizes are listed in table 2.

**Table 2.** Zinc content and weight for different particle sizes.

particle size (μm)	raw material	+380	380–250	250–180	180–150	150–120	120–109	109–96	+96
wt/%	100	6.54	8.60	8.17	5.69	36.21	11.18	17.94	5.68
Zn/%	24.74	23.24	24.20	24.70	24.64	24.33	24.74	24.14	23.33

**Figure 3.**
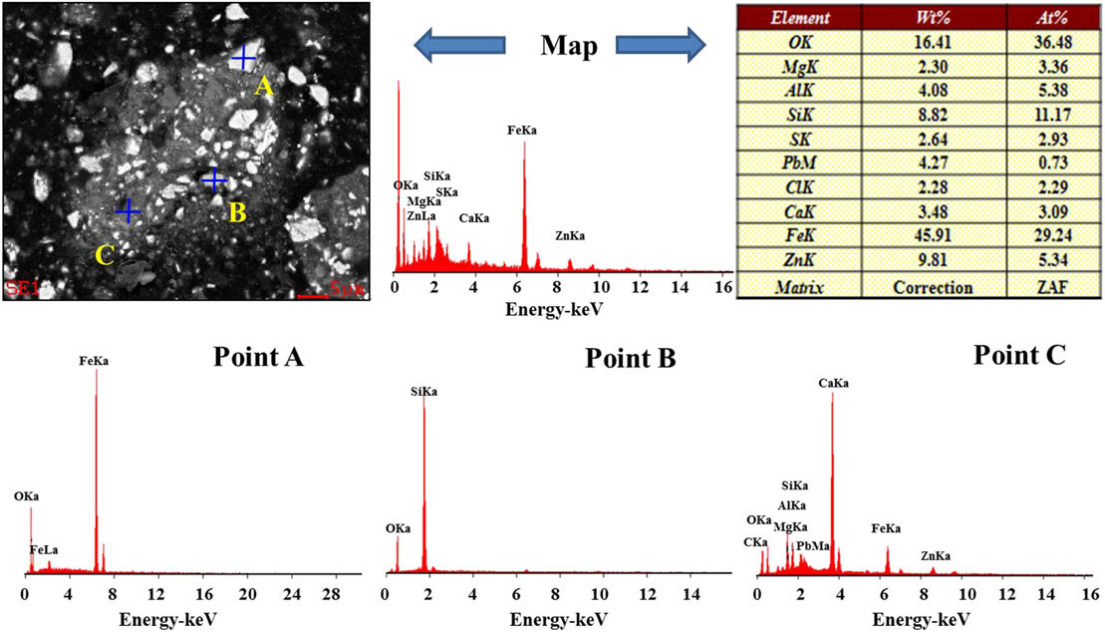
SEM-ESD pattern of the MSD.

**Figure 4.**
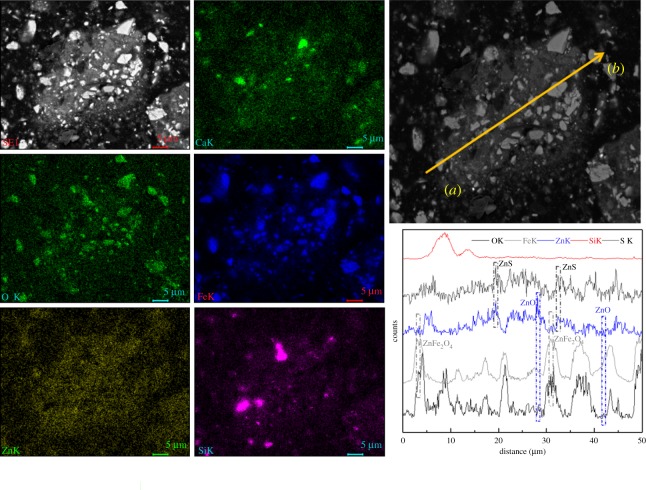
SEM-EDS maps and line-scanning pattern of the MSD.

Scanning electron microscopy (SEM; using an XL30ESEM-TMP, Philips Company, The Netherlands) with energy-dispersive X-ray spectroscopy (EDS; using a GENESIS, EDAX Company, USA) measurements of MSD sample particles were performed to obtain additional information on the structure, morphology and chemical composition of the MSD, as shown in figures 3 and 4. Figure 3 shows that the dust mainly exists in two typical structures: granular (points A and B) and amorphous (point C) structures. The granular structures are mainly iron oxide (point A) and gangue minerals (point B), and Zn with Fe, O, Pb, Ca, Si and Al is primarily found in the amorphous structure. From figure 4, we found that the zinc exists in the form of ZnO, Zn_2_SiO_4_, ZnS and ZnFe_2_O_4_. On the other hand, the zinc was encapsulated by iron, gangue and other minerals.

### 2.2. Experimental methods

**Figure 5.**
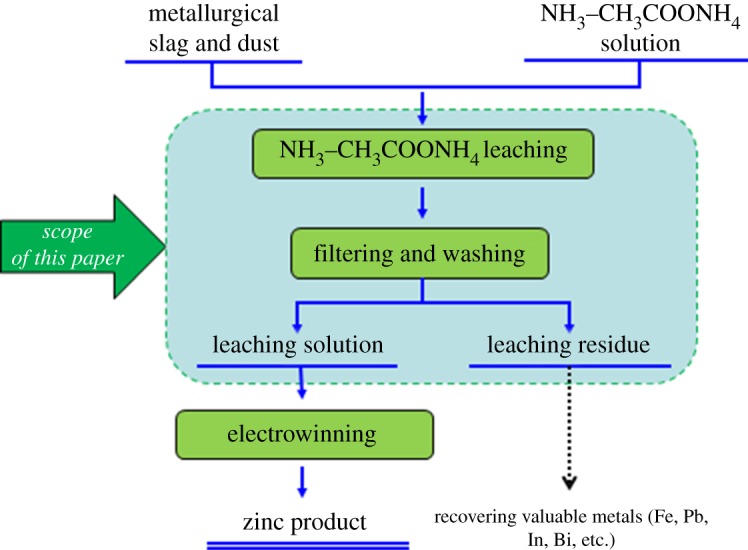
Flow diagram of the leaching process for MSD.

All reagents used in this study were either of chemical or analytical grade. The leaching experiment was conducted in a 300 ml closed glass reactor equipped with a magnetic stirrer to mix the MSD sample with the leaching agent. The flow diagram of the zinc leaching process obtained on adding 20 g of a sample to the Erlenmeyer flask is shown in figure 5. The parameters of the leaching experiments are as follows: temperature, reaction time, stirring speed, solid/liquid ratio, total ammonia concentration and ammonia/ammonium ratio. In all the experiments, the zinc dissolution concentration was determined using the EDTA titrimetric method.

After the leaching experiments, the leaching recovery of zinc (*η*_Zn_, %) was calculated according to the following equation:

$$\eta_{\mathrm{Zn}}=\frac{C_{\mathrm{Zn}}\times V}{m\times w_{\mathrm{Zn}}^{*}},$$

where *C*_Zn_ is the Zn concentration of the leaching solution (g l^−1^), *V* is the leaching solution volume (l), *m* is the mass of the MSD (g) and $w_{\mathrm{Zn}}^{*}$ is the Zn content of the MSD (%).

### 2.3. Characterization methods

The vibrational spectra of the synthesized compound using the Fourier transform infrared (FT-IR; Thermo Scientific Nicolet iS50, USA) spectroscopic technique were recorded to obtain more information on the mechanisms of zinc extraction from the MSD in the NH_3_–CH_3_COONH_4_–H_2_O system. The FT-IR spectra in the region 400–4000 cm^−1^ were recorded using the KBr pellet technique.

To explicitly define the formula and structural formula of the Zn complexes in the leaching process, electrospray ionization mass spectrometry (ESI-MS) experiments were performed in the positive mode using a Bruker micrOTOF-Q II™ ESI-Qq-TOF mass spectrometer, with *m/z* between 50 and 700. ESI-MS is a technique that allows the ions in the solution to be transformed into the gas phase, where they can be analysed and eventually characterized to provide maximum certainty in small molecule identification [26,27]. The easy determination of the formula of small molecules and analyses of complex mixtures are key applications of ESI-MS.

## 3. Results and discussion

### 3.1. Leaching reaction of zinc extraction process

In NH_3_–CH_3_COONH_4_–H_2_O solutions, the dissolved zinc oxide (ZnO) can combine with ammonium ions and ammonia to form soluble [Zn(NH_3_)*_i_*]^2+^ complexes, as shown in equations (3.1) and (3.2):

3.1 $$\text{ZnO}+i\text{N}{{\text{H}}_{4}}^{+}={\left[\text{Zn}{\left(\text{N}{\text{H}}_{3}\right)}_{i}\right]}^{2+}+{\text{H}}_{2}\text{O}+(i-2){\text{H}}^{+}$$

and

3.2 $$\text{ZnO}+i\text{N}{\text{H}}_{3}+{\text{H}}_{2}\text{O}=\text{Zn}{\left({\text{NH}}_{\text{3}}\right)}_{i}^{2+}+2\text{OH}.$$

In addition, for the leaching system of ZnO–RCOOH–H_2_O [23,25], the carboxylate anion (RCOO^−^) plays an important role in the zinc leaching process, zinc ions can combine with carboxyl groupings to form stable complexes, and the zinc extraction might be better explained using substituent group effects from the Lewis acid/base theory, as shown in the following equation:

3.3 $$\text{2RCO}{\text{O}}^{-}+\text{ZnO}\overset{\left({\text{H}}_{3}{\text{O}}^{+}\right)}{⟷}{\text{(RCOO)}}_{2}\text{Zn}+{\text{H}}_{2}\text{O}.$$

Based on a combination of theory and practical applications from the industrial prospective, seven key parameters, namely particle size, stirring speed, liquid/solid ratio, total ammonia concentration, ammonia/ammonium ratio $\left([\text{N}{\text{H}}_{3}]/[\text{N}{{\text{H}}_{4}}^{+}]\right)$, leaching temperature and leaching time, were studied.

#### 3.1.1. Effects of particle size

**Figure 6.**
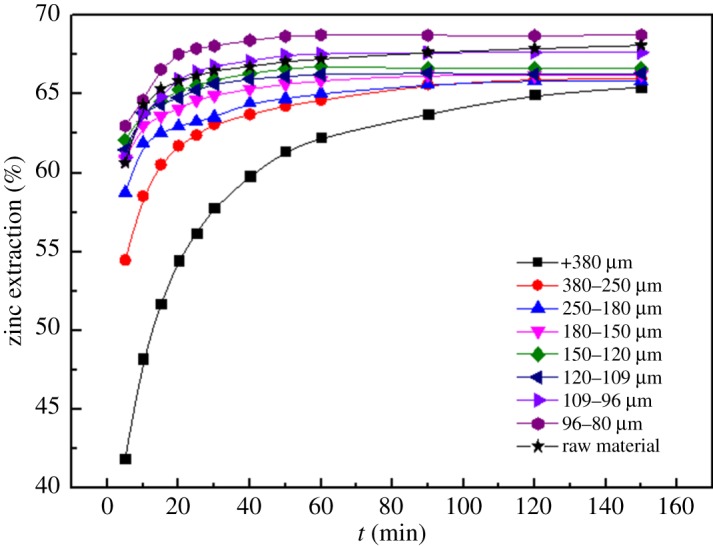
Effect of particle size on zinc extraction.

The effect of the particle sizes on the Zn leaching efficiency was determined as a function of time and shown in figure 6. The other parameters that were maintained constant during the leaching process were total ammonia concentration of 4 mol l^−1^, temperature of 25°C, reaction time of 2.5 h, stirring speed of 300 r.p.m., solid/liquid ratio of 1 : 3 and ammonia/ammonium ratio of 1 : 1. The zinc extraction efficiency for a variety of particle sizes was compared with that of the raw material.

The research results show that the decrease in particle size significantly increases the zinc extraction. This is important because the fine particle size allows the existence of a greater number of active sites and promotes the contact between the ore surface with the reactants, which leads to greater zinc extraction [24]. At the same time, we found that the zinc extraction for the raw material rises from 60.63% to 67.23% when the leaching time is increased from 5 min to 60 min. However, the zinc extraction for the raw material (67.23%) is almost the same as that for 150–120 µm (66.77%), 120–109 µm (66.25%), 109–96 µm (67.55%) and 96–80 µm (68.75%) in 60 min. In general, considering the economic feasibility, the raw material should be used in follow-on experiments.

#### 3.1.2. Effects of stirring speed

**Figure 7.**
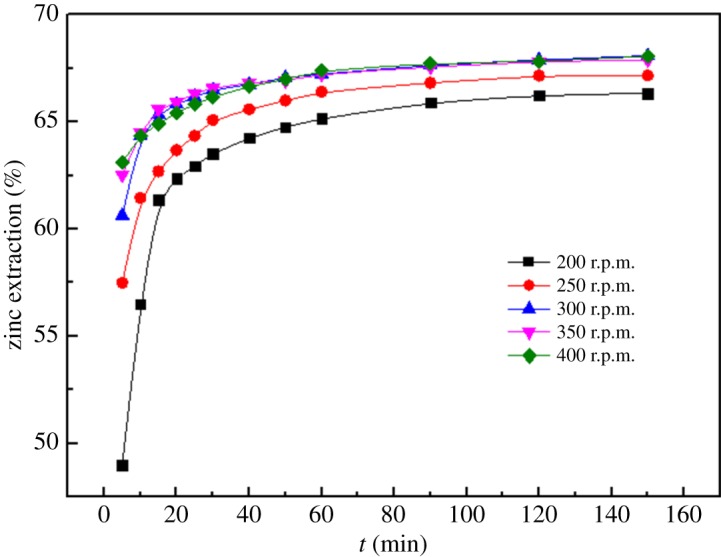
Effect of different stirring speeds on leaching rate of zinc.

The effects of different stirring speeds on the dissolution of zinc from the MSD are shown in figure 7. The effects of five stirring speeds were studied in the experiments with a total ammonia concentration of 4 mol l^−1^, temperature of 25°C, reaction time of 2.5 h, solid/liquid ratio of 1 : 3 and ammonia/ammonium ratio of 1 : 1. The stirring speed has a significant effect on the dissolution of zinc; as the stirring speed increases from 200 r.p.m. to 300 r.p.m., the zinc extraction increases from 49.00% to 60.63% in 5 min. As the stirring speed increases, the improvement in the zinc extraction promotes the diffusion of the reactants. However, for a further increase in the stirring speed above 300 r.p.m., the zinc extraction remains approximately constant from 10 min to 150 min. Therefore, the stirring speed was determined at 300 r.p.m. in subsequent experiments.

#### 3.1.3. Effects of liquid/solid ratio

**Figure 8.**
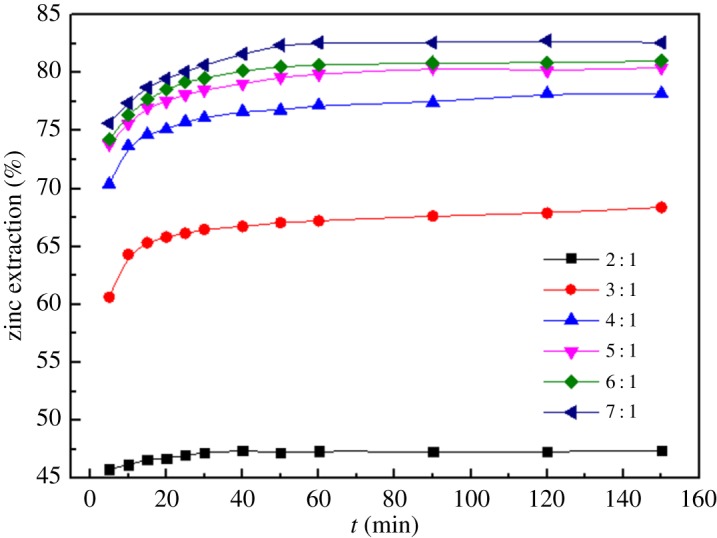
Effect of different liquid/solid ratios on leaching rate of zinc.

Figure 8 describes the effects of the liquid/solid ratios on the dissolution of the MSD in the range of 2 : 1–7 : 1 with a stirring speed of 300 r.p.m., total ammonia concentration of 4 mol l^−1^, temperature of 25°C, reaction time of 2.5 h and ammonia/ammonium ratio of 1 : 1. It can be observed from the figure that by increasing the liquid/solid ratio from 2 : 1 to 4 : 1, the leaching rate of zinc significantly increases from 47.35% to 77.21% after 60 min of leaching. An increase in the liquid/solid ratio causes the initial concentration of the reaction reagents to also increase correspondingly and leads to a greater mass transfer driving force. Hence, this result promotes the formation of Zn complexes. However, the increase in the zinc extraction is very slow as the liquid/solid ratio continues to increase from 4 : 1 to 7 : 1. Therefore, using a greater liquid/solid ratio leads to high cost, and an appropriate liquid/solid ratio of 4 : 1 is thus selected for further experiments.

#### 3.1.4. Effects of total ammonia concentration

**Figure 9.**
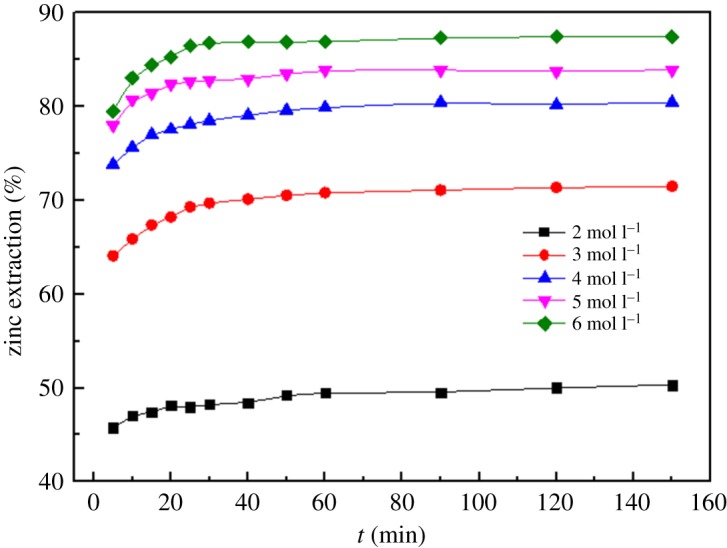
Effect of different total ammonia concentrations on leaching rate of zinc.

To investigate the effect of the total ammonia concentration on the extraction of zinc, the total ammonia concentration was varied from 2 mol l^−1^ to 6 mol l^−1^ at a temperature of 25°C, reaction time of 2.5 h, stirring speed of 300 r.p.m., solid/liquid ratio of 1 : 5 and ammonia/ammonium ratio of 1 : 1. The results are shown in figure 9.

Figure 9 shows that the extraction efficiencies of Zn increase from 49.49% to 83.86% after 60 min of leaching with the increase in the total ammonia concentration from 2 mol l^−1^ to 5 mol l^−1^. The results suggest that a higher total ammonia concentration leads to a more effective extraction of Zn; however, this is not always the case. An increase in the total ammonia concentration from 5 mol l^−1^ to 6 mol l^−1^ had no significant effect on the zinc recovery, i.e. it only showed a 3.02% increase. Considering the economic feasibility, the total ammonia concentration should be as low as possible when the extraction efficiency of Zn reaches saturation. Thus, an appropriate total ammonia concentration of 5 mol l^−1^ is favourable for higher productivity in the experiment.

#### 3.1.5. Effects of ammonia/ammonium ratio

**Figure 10.**
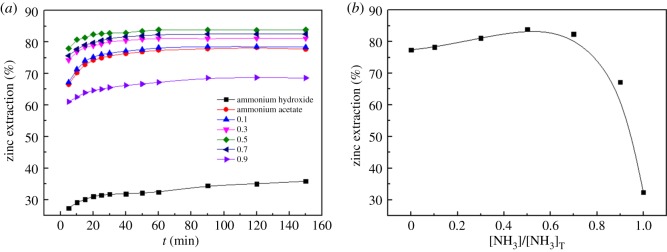
Effect of various total ammonia concentrations on leaching rate of zinc: (*a*) time difference; (*b*) 60 min of leaching.

To investigate the effect of the ammonia/ammonium ratio $\left([\text{N}{\text{H}}_{3}]/[\text{N}{{\text{H}}_{4}}^{+}]\right)$ on the zinc extraction, experiments were performed in the NH_3_–CH_3_COONH_4_–H_2_O system for various ammonia/ammonium ratios under the following conditions: stirring speed of 300 r.p.m., total ammonia concentration of 5 mol l^−1^, temperature of 25°C, reaction time of 2.5 h and solid/liquid ratio of 1 : 5. At the same time, the NH_3_–H_2_O system and CH_3_COONH_4_–H_2_O system were compared with the NH_3_–CH_3_COONH_4_–H_2_O system under the same conditions. The results are shown in figure 10. It shows that the effective extraction of Zn first increased and then decreased with an increase in the ammonia/ammonium ratio from 1 : 9 ([NH_3_]/[NH_3_]_T _= 0.1) to 9 : 1 ([NH_3_]/[NH_3_]_T _= 0.9) in the NH_3_–CH_3_COONH_4_–H_2_O system (figure 10*b*). And the leaching rate of zinc reached the maximum value (83.86%) after 60 min of leaching when the ammonia/ammonium ratio was 1 : 1 ([NH_3_]/[NH_3_]_T _= 0.5). The results also suggest that the leaching rate of zinc in the NH_3_–H_2_O system (32.45%) and CH_3_COONH_4_–H_2_O system (77.40%) is relatively lower than that in the NH_3_–CH_3_COONH_4_–H_2_O system (83.86%). In NH_3_–H_2_O solutions, the predominant zinc species are [Zn(NH_3_)_*i*_]^2+^ complexes. The addition of CH_3_COONH_4_ to the NH_3_–H_2_O solutions transforms the original [Zn(NH_3_)_*i*_]^2+^ complexes into the more stable [Zn(CH_3_COO)(NH_3_)_*i*_]^+^ complexes, which further promotes the dissolution of insoluble zinc minerals. The result further shows that the addition of ammonium acetate plays a positive role in the dissolution process of the MSD.

#### 3.1.6. Effects of leaching temperature

**Figure 11.**
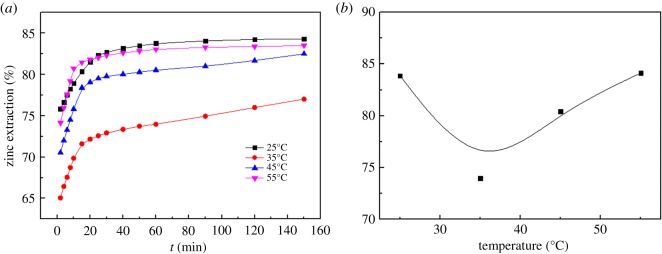
Effect of various leaching temperatures on leaching rate of zinc: (*a*) time difference; (*b*) 60 min of leaching.

The effects of different temperatures on the zinc extraction were studied for different leaching times using a 5 mol l^−1^ total ammonia concentration with a stirring speed of 300 r.p.m., solid/liquid ratio of 1 : 5 and ammonia/ammonium ratio of 1 : 1. The results are shown in figure 11.

As can be observed from figure 11, the leaching efficiencies of the zinc have an apparent dependence on the leaching temperature. The leaching efficiencies of zinc exhibit an apparent increase as the temperature increases in 30 min. It is worth noting that the effective extraction of Zn is first decreased and then increased with the increase in the temperature from 25°C to 55°C after 60 min of leaching, and the leaching rate of zinc at 35°C (73.96%) is significantly less than that at 25°C (83.76%) and 55°C (83.04%), as shown in figure 11*b*.

The aforementioned observations can be explained as follows: the energy available for atomic and molecular collisions increases, and the mass transfer coefficient, reaction constant and diffusivity are all improved with an increase in the temperature. We hypothesize that the reaction constant and diffusivity have little influence on the leaching efficiencies of zinc; however, the effect of solvent evaporation is dominant in the range of 25–35°C. The ammonia/ammonium ratio is decreased with the increase in the ammonia volatility, which results in the decreased leaching efficiencies of zinc. However, the effects of the reaction constant and diffusivity on the leaching efficiencies of zinc are much greater than that of the solvent evaporation with the increase in the reaction temperature from 35°C to 55°C. Therefore, it also enhances the reaction speed and significantly shortens the reaction time.

Therefore, considering economic feasibility, an appropriate leaching temperature of 25°C is considered to be optimal to facilitate the zinc extraction (83.76%).

### 3.2. Characterization analysis

#### 3.2.1. Leaching solution analysis

**Figure 12.**
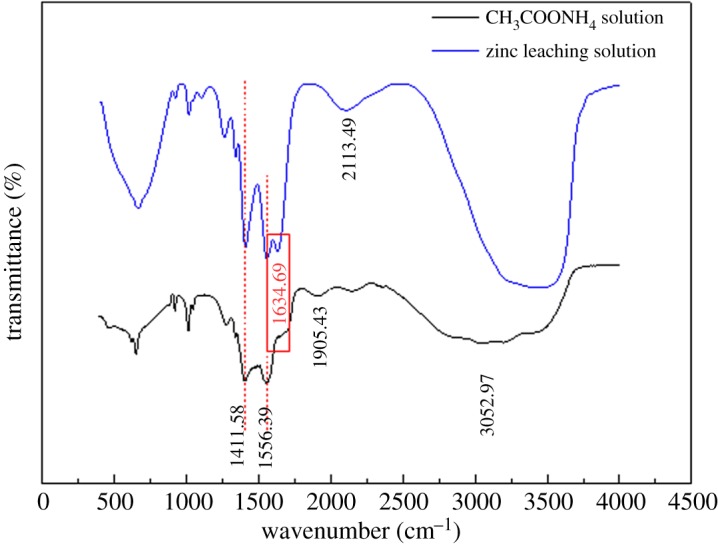
FT-IR spectra of CH_3_COONH_4_ solution and zinc leaching solution.

To obtain more information on the mechanisms of zinc extraction from the MSD in a NH_3_–CH_3_COONH_4_–H_2_O system, we studied the vibrational spectra of the synthesized compound using FT-IR spectroscopic techniques. The experimental IR spectra of the CH_3_COONH_4_ solution and zinc leaching solution are shown in figure 12.

The FT-IR spectrum exhibits strong absorptions for the carbonyls of the carboxylate ligands in the asymmetric stretching vibrations *υ*_as_ (COO^−^) and the symmetric stretching vibrations *υ*_s_ (COO^−^). Asymmetric stretching vibrations *υ*_as_ (COO^−^) appear between 1607 and 1660 cm^−1^, whereas the symmetric vibrations *υ*_s_ (COO^−^) are observed between 1537 and 1380 cm^−1^ [22,28].

Figure 12 shows that three typical FT-IR peaks centred at approximately 1411.58, 1556.39 and 1634.69 cm^−1^ associated with the stretching and bending frequencies of complex species were found in agreement with previously examined complexes of citrates with various metals. In the leaching solution of zinc, the bands are shifted to higher frequencies (1634.69 cm^−1^) compared to the free CH_3_COONH_4_, which indicates the changes in the vibrational status of the ligand upon complexation to Zn(II).

#### 3.2.2. Mass spectrometric analysis

Based on the aforementioned analysis, the substituent groups attached to the carboxylic acid functionality can play an important role in the zinc extraction process. All of the carboxylates could be employed in coordination with the zinc ion(s) thus possibly improving the stability of the zinc complexes composed of zinc ions and the ligand. At the same time, the formula and structural formula of the Zn complexes in the leaching solution were obtained using the ESI-MS analysis.

**Figure 13.**
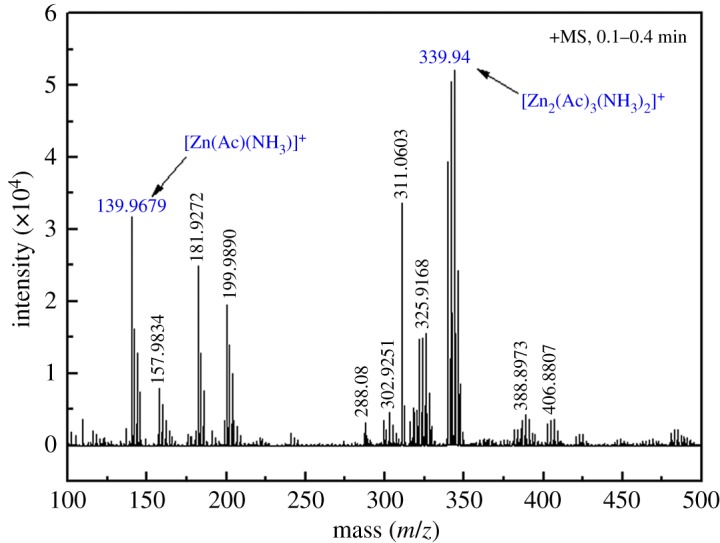
ESI-MS analysis of Zn complexes in positive mode (total ammonia concentration of 5 mol l^−1^, stirring speed of 300 r.p.m., solid/liquid ratio of 1 : 4, ammonia/ammonium ratio of 1 : 1 and leaching temperature of 25°C).

The mass spectra of the Zn complexes in the positive mode are shown in figure 13. Table 3 summarizes the data of the Zn complexes identified in this study, including peak number, molecular formula and fragment ions. As suggested in figure 13 and table 3, it is also important to know that a wide variety of Zn complexes exist in the leaching solution. At the same time, we found that peak 7 (*m/z* 339.94) is the strongest peak. In addition, the strongest peak reveals a mass error of less than 5 ppm (2.7 ppm) for reading out a formula by smart-formula scoring. The results provide a superior smart-formula scoring for enhanced confidence in the structural analysis of the Zn complexes.

**Table 3.** Mass spectral data (molecular and fragment ions) of the main Zn complexes.

peak	+meas. *m/z*	formula
1	139.9776	[Zn(Ac)(NH_3_)]^+^
2	157.9834	[Zn(Ac)(NH_3_)_2_]^+^
3	181.9791	Zn(Ac)_2_
4	199.9902	[Zn(Ac)_2_(NH_3_)]^+^
5	304.8969	[Zn_2_(Ac)_3_]^+^
6	321.9247	[Zn_2_(Ac)_3_(NH_3_)]^+^
7	339.9366	[Zn_2_(Ac)_3_(NH_3_)_2_]^+^

**Figure 14.**
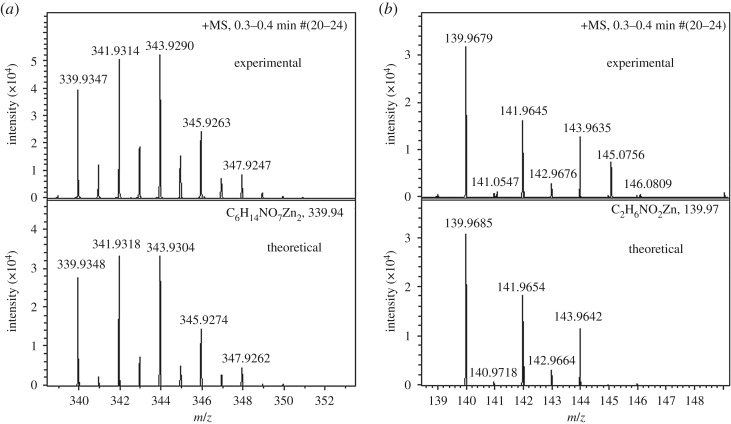
Comparison of experimental isotopic distribution and theoretical isotopic distribution simulated by Bruker Xmass 6.1.2 software at the peaks of (*a*) 139.9679 and (*b*) 339.9347.

The measured spectra of the Zn complexes of peak 1 (*m/z* 139.97) and peak 7 (*m/z* 339.94) are matched to a Bruker database in figure 14*a*,*b* as simulated by the Bruker Xmass 6.1.2 software. The results showed a high degree of matching of the experimental and theoretical spectra. On the basis of the mass spectral data, these relatively stable Zn complexes were tentatively identified as [Zn_2_(Ac)_3_(NH_3_)_2_]^+^.

#### 3.2.3. Solid residue analysis

**Figure 15.**
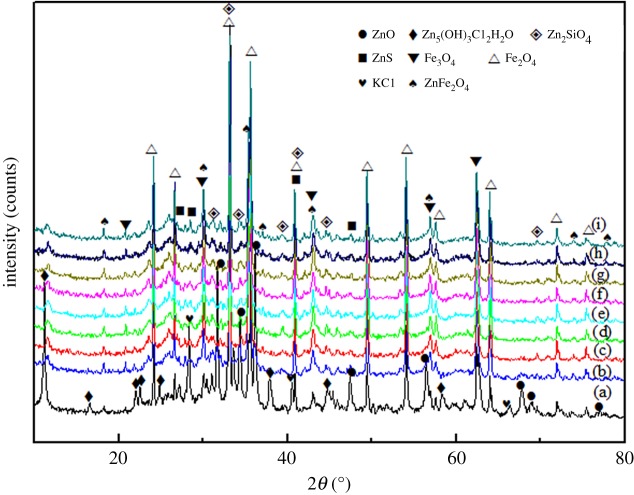
XRD patterns of leaching slags formed for various leaching times: (a) raw material, (b) 2 min, (c) 4 min, (d) 6 min, (e) 8 min, (f) 10 min, (g) 20 min, (h) 30 min and (i) 40 min.

To understand the solubility behaviour of various zinc phases, typical XRD patterns of the leaching residue for different leaching times are shown in figure 15. The optimal experimental conditions for leaching MSD are obtained for the following conditions: total ammonia concentration of 5 mol l^−1^, stirring speed of 300 r.p.m., solid/liquid ratio of 1 : 5, ammonia/ammonium ratio of 1 : 1 and leaching temperature of 25°C. Figure 15 shows that the diffraction peaks of ZnO and Zn_5_(OH)_8_Cl_2_H_2_O disappear very quickly and the main compositions of the residue were zinc ferrite (ZnFe_2_O_4_) and iron oxides (Fe_3_O_4_, Fe_2_O_3_).

**Figure 16.**
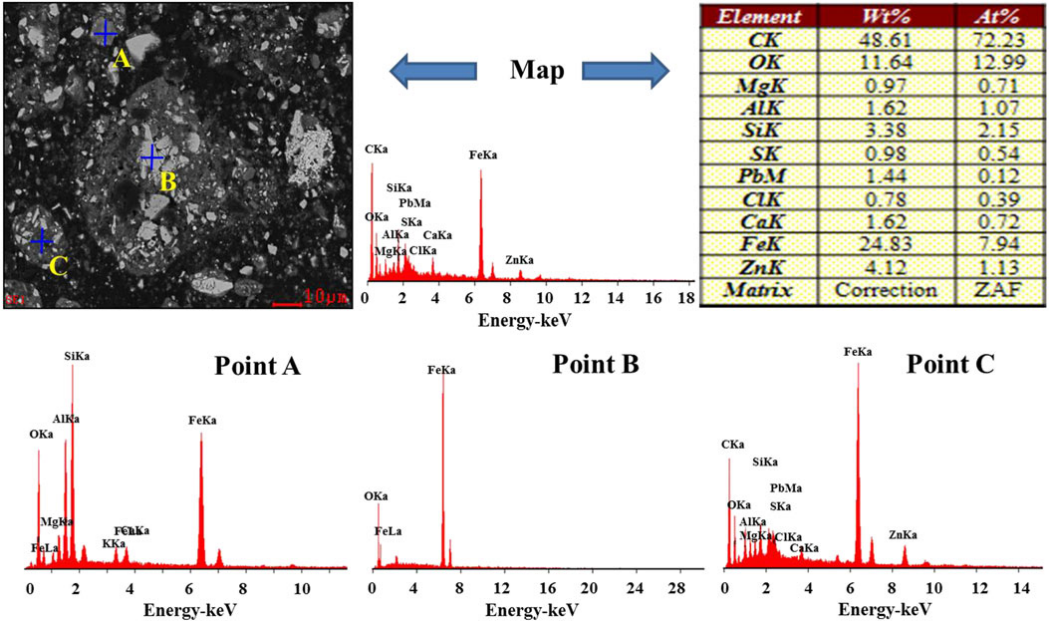
SEM-ESD pattern of MSD leaching residue.

**Figure 17.**
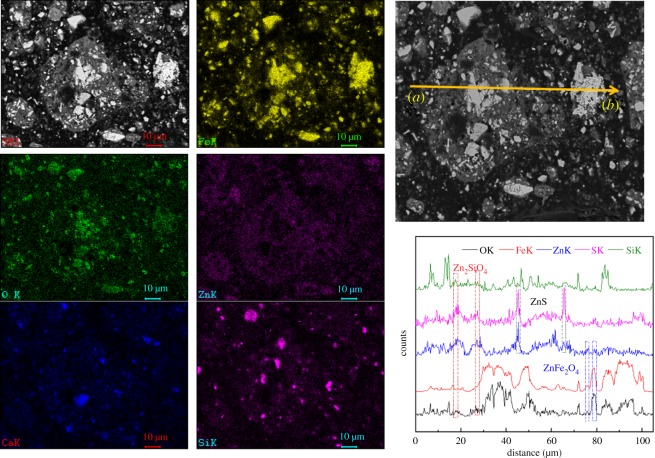
SEM-EDS maps and line-scanning pattern of MSD leaching residue.

To further investigate the particle distribution, size and composition of the leaching residue in 40 min, the leaching residue was characterized using SEM-EDS, as shown in figures 16 and 17. Figure 16 shows that the structure and particle configuration of the residue, and the presence of C, O, Si, S, Ca, Fe, Mg, Al, Cl and Zn are relevant to the investigated system. The EDS maps of Ca, O, Fe, Si and Zn were analysed as shown in figure 17. The SEM-EDS surface scanning pattern shows that the residue comprises a metallic mineral phase in addition to a gangue mineral phase. Furthermore, the SEM-EDS line-scanning pattern of the MSD leaching residue was also obtained as shown in figure 17. The line-scanning pattern makes it clear that Zn_2_SiO_4_, ZnS and ZnFe_2_O_4_ are not leaching in the NH_3_–CH_3_COONH_4_–H_2_O system.

### 3.3. Kinetic analysis

To determine the kinetic parameters and the rate-controlling step for the dissolution of the MSD in the NH_3_–CH_3_COONH_4_–H_2_O system, the rate of MSD dissolution is tested against a shrinking core model. The shrinking core model considers that the leaching process is controlled either owing to the diffusion of the reactant through the solution boundary layer or a solid product layer, or owing to the rate of the surface chemical reaction [29].

Assuming that the MSD particles have a spherical geometry and the process is controlled by the diffusion reaction, the integrated rate equation of the shrinking core model can be expressed as follows:

3.4 $$k_{d}\cdot t=\frac{1-2}{3x}-{\left(1-x\right)}^{2/3}.$$

When the process is controlled by the chemical reaction, it will have an integrated rate equation as follows:

3.5 $$k_{r}\cdot t=1-{\left(1-x\right)}^{1/3},$$

where *x* is the reaction fraction, *k*_d_ and *k*_r_ are the rate constants and *t* is the reaction time.

In addition, an empirical model suggested by Dickinson & Heal [30] is used to describe the dissolution process. This model is based on the assumption that both the interface transfer and diffusion through the product layer affect the leaching rate.

The equation for this model is given as follows:

3.6 $$k_{0}\cdot t=\frac{1}{3}\text{ln}(1-x)-[1-{\left(1-x\right)}^{-1/3}],$$

where *k*_0_ is the rate constant for mixed control.

**Figure 18.**
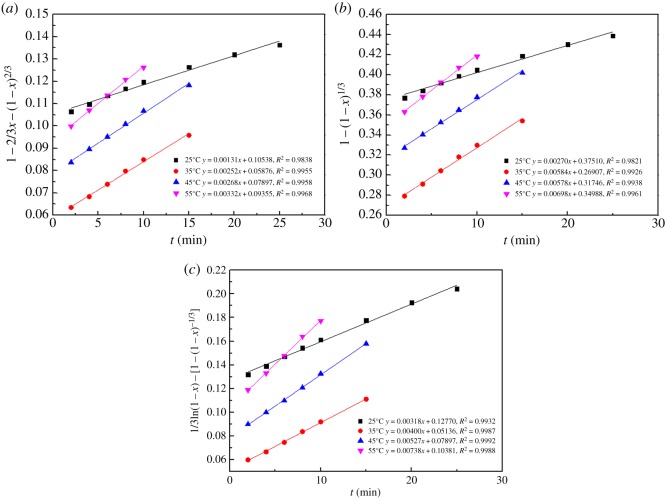
Plot of (*a*) 1 − 2/3*x* − (1 − *x*)^2/3^, (*b*) 1 − (1 − *x*)^1/3^ and (*c*) 1/3ln(1 − *x*) − [1 − (1 − *x*)^−1/3^] versus time for various temperatures.

The plots of 1–2/3*x* − (1 − *x*)^2/3^, 1 − (1 − *x*)^1/3^, and 1/3ln(1 − *x*) − [1 − (1 − *x*)^−1/3^] versus the time *t* for different temperatures are given in figure 18. At the same time, the fitting equations of 1–2/3*x* − (1 − *x*)^2/3^, 1 − (1 − *x*)^1/3^, and 1/3ln(1 − *x*) − [1 − (1 − *x*)^−1/3^] are given for various temperatures. The correlation coefficients of the fitting results for the various models at the various temperatures are shown in table 4. It is evident that the mathematic model of equation (3.6) was adopted for nonlinear least-squares data fitting to process the experimental data, and a high correlation coefficient of 0.99 was obtained, which is greater than that obtained with the other two models of equations (3.4) and (3.5). In addition, the temperature dependence of the reaction rate constant was calculated using the Arrhenius equation:

3.7 $$k=A\cdot \text{exp}\left(\frac{-E_{a}}{RT}\right),$$

where *A* is the frequency factor, *E*_a_ is the activation energy of the reaction (kJ mol^−1^), *R* is the universal gas constant and *T* is the absolute temperature (K).

**Table 4.** Correlation coefficients of fitting results for various models at various temperatures.

	*R*^2^		
*T*	1 − 2/3*x* − (1 − *x*)^2/3^	1 − (1 − *x*)^1/3^	1/3ln(1 − *x*) − [1 − (1 − *x*)^−1/3^]
25°C	0.9838	0.9821	0.9932
35°C	0.9955	0.9926	0.9987
45°C	0.9958	0.9938	0.9992
55°C	0.9968	0.9961	0.9988

**Figure 19.**
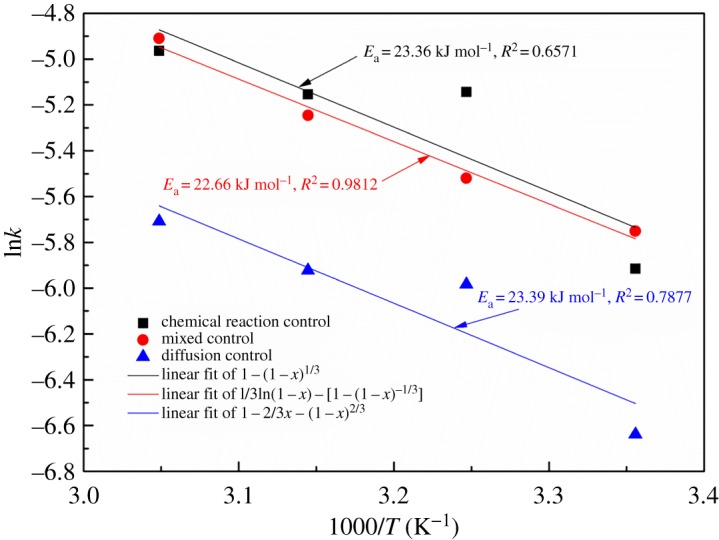
Arrhenius curves obtained for the dissolution of MSD.

The Arrhenius curves of ln*k* versus 1000/*T* for different models are shown in figure 19. Figure 19 presents a good fit (*R*^2 ^> 0.9812). The calculated activation energy for the dissolution of the MSD is 22.66 kJ mol^−1^. The result matches with that of recent research on the dissolution of low-grade oxidized copper ore (22.91 kJ mol^−1^) [31] in NH_3_–(NH_4_)_2_SO_4_ solutions, leaching of malachite in NH_3_–(NH_4_)_2_SO_4_ solutions (26.75  kJ mol^−1^) [32] and leaching of low-grade zinc oxide ores (22.30  kJ mol^−1^) in NH_4_Cl–NH_3_ solutions with NTA [24].

Consequently, these values clearly confirm that the dissolution process can be intensified and that the leaching process, i.e. leaching rate, is controlled by both the interfacial transfer and diffusion across the product layer.

## 4. Conclusion

In this study, investigations were conducted into a method for improving the zinc recovery from MSD in a NH_3_–CH_3_COONH_4_–H_2_O system. The total ammonia concentration, liquid/solid ratio and ammonia/ammonium ratio had a significant effect on the zinc recoveries. The experimental results indicated that the zinc extraction reached 83.76% when using a total ammonia concentration of 5 mol l^−1^, stirring speed of 300 r.p.m., ammonia/ammonium ratio of 1 : 1 and solid/liquid ratio of 1 : 5 for 60 min at 25°C. The dissolution kinetics of the MSD in the NH_3_–CH_3_COONH_4_–H_2_O solution was controlled by the interface transfer and diffusion and the apparent activation energy of the reaction was 22.66 kJ mol^−1^.

Moreover, the zinc oxide could combine with the carboxylate anion to form relatively stable Zn complexes such as [Zn_2_(Ac)_3_(NH_3_)_2_]^+^. Zinc ferrite (ZnFe_2_O_4_), iron oxides (Fe_3_O_4_ and Fe_2_O_3_) and gangue minerals were insoluble materials in the NH_3_–CH_3_COONH_4_–H_2_O system. In addition, Zn_2_SiO_4_, ZnS and ZnFe_2_O_4_ did not leach in the NH_3_–CH_3_COONH_4_–H_2_O system, which was one of the main causes of the lower zinc extraction rate.

## Supplementary Material

Date

## Data accessibility

The datasets supporting this article have been uploaded as part of the electronic supplementary material.
